# Pain frequency moderates the relationship between pain catastrophizing and pain

**DOI:** 10.3389/fpsyg.2014.01421

**Published:** 2014-12-19

**Authors:** Heidi Kjøgx, Robert Zachariae, Mogens Pfeiffer-Jensen, Helge Kasch, Peter Svensson, Troels S. Jensen, Lene Vase

**Affiliations:** ^1^Department of Psychology and Behavioural Sciences, School of Business and Social Sciences, Aarhus UniversityAarhus, Denmark; ^2^MindLab, Center for Functionally Integrative Neuroscience, Aarhus University HospitalAarhus, Denmark; ^3^Unit for Psychooncology and Health Psychology, Department of Oncology, Aarhus University Hospital and Department of Psychology and Behavioural Science, Aarhus UniversityAarhus, Denmark; ^4^Department of Rheumatology, Aarhus University HospitalAarhus, Denmark; ^5^Department of Neurology, Aarhus University HospitalAarhus, Denmark; ^6^Section of Clinical Oral Physiology, School of Dentistry, Aarhus UniversityAarhus, Denmark; ^7^Danish Pain Research Center, Aarhus University HospitalAarhus, Denmark

**Keywords:** frequency, pain catastrophizing scale, depression, anxiety, danish validation

## Abstract

**Background:** Pain frequency has been shown to influence sensitization, psychological distress, and pain modulation. The present study examined if pain frequency moderates the relationship between pain catastrophizing and pain.

**Method:** A non-clinical (247 students) and a clinical (223 pain patients) sample completed the Danish versions of the Pain Catastrophizing Scale (PCS), Beck Depression Inventory, and the State Trait Anxiety Inventory and rated pain intensity, unpleasantness and frequency.

**Results:** In both samples, high pain frequency was found to moderate the association between pain catastrophizing and pain intensity, whereas low pain frequency did not. The psychometric properties and the factor structure of the Danish version of the PCS were confirmed.

**Conclusions:** This is the first study to validate the Danish version of the PCS and to show that pain frequency moderates the relationship between pain catastrophizing and reported pain in both non-clinical and clinical populations.

## Introduction

Pain catastrophizing is defined as a negative cognitive-affective response to anticipated or actual pain (Sullivan et al., 1995) and has been shown to account for up to 31% of the variance in pain ratings (Severeijns et al., 2001; Sullivan et al., 2001; Edwards et al., 2011; Flor and Turk, 2011; Vase et al., 2012). Conceptually and empirically, pain catastrophizing shows considerable overlap with anxiety and depressive symptoms, so to test whether it is a unique predictor of pain, associations between pain catastrophizing and pain should be controlled for levels of anxiety and depressive symptoms (Quartana et al., 2009; Khan et al., 2012; Wade et al., 2012).

Across non-clinical and clinical studies, pain catastrophizing has been shown to contribute significantly to pain levels (Beneciuk et al., 2010; Vranceanu et al., 2010; Lucey et al., 2011). However, studies examining the unique contribution of pain catastrophizing, i.e., independently of anxiety and/or depressive symptoms, to reported pain ratings have shown mixed results, both when comparing non-clinical and clinical samples as well as comparing within clinical samples (Sullivan et al., 1995; Crombez et al., 2003; Granot and Ferber, 2005; Katz et al., 2009; Meyer et al., 2009; Keogh et al., 2010; Vase et al., 2011). The varying results do not appear to be related to the type or intensity of pain or to the level of pain catastrophizing nor to common confounding factors such as age, gender and the size of the study (Kjøgx et al., in preparation). One factor that may influence the relationship between pain catastrophizing and pain ratings is pain frequency. Experimental and clinical studies have documented that repetitive noxious stimulation contributes to enhanced pain perception (Vase et al., 2011; Woolf, 2011). In addition, higher pain frequency has been found related to increased sensitization (Buchgreitz et al., 2008), increased levels of anxiety, depression, and distress (Kuch et al., 1993; Spiegel et al., 1994; Fishbain et al., 1997; Zwart et al., 2003; Cathcart et al., 2010) and altered pain modulation (Price and Vase, 2013). As clinical populations generally experience not only higher pain levels, but also a higher frequency of pain compared to non-clinical populations, and at the same time increased levels of psychological symptoms such as anxiety and depression (Derogatis et al., 1983), it can be speculated that pain frequency mediates the relationship between psychological factors and pain. Thus, it can be hypothesized that pain frequency may play a greater role for the association between pain catastrophizing and pain in clinical rather than non-clinical populations. Still, it may also influence the relationship between pain catastrophizing and pain in non-clinical populations with a high pain frequency.

Nevertheless, the influence of pain frequency on the unique contribution of pain catastrophizing to pain ratings, independently of anxiety, and depressive symptoms, has not yet been tested in non-clinical and clinical pain populations.

Pain catastrophizing is usually measured with the Pain Catastrophizing Scale (PCS) (Sullivan et al., 1995). However, so far the PCS has not been validated in a Danish population, and to ensure the validity of the PCS in Danish non-clinical and clinical populations, the psychometric properties of the Danish version of the PCS need to be evaluated.

In the present study, pain catastrophizing, anxiety, depression, pain intensity, unpleasantness, and frequency were assessed in a non-clinical sample (students) and a clinical sample (rheumatoid arthritis and headache patients). In addition, we validated the use of the PCS for assessing pain catastrophizing in Danish non-clinical and clinical pain samples.

We hypothesized that high levels of pain catastrophizing would contribute to pain independently of anxiety and depressive symptoms and that high levels of pain frequency would moderate the relationship between pain catastrophizing and pain levels independently of anxiety and depressive symptoms.

## Materials and methods

### Participants

Two groups of participants were investigated: a non-clinical sample of 247 university students and a clinical sample of 223 pain patients. The non-clinical sample included 153 undergraduate students enrolled at the Department of Psychology and Behavioral Sciences, Aarhus University (third semester), and 94 undergraduate students attending the Engineering College of Aarhus (second semester). The students completed the questionnaires after class. The clinical sample consisted of two groups of chronic pain patients: 113 patients with rheumatoid arthritis recruited from the Department of Rheumatology, Aarhus University Hospital, during follow-up appointments, where they received written information about the study and were invited to participate, and 110 headache patients recruited from the Headache Clinic at the Department of Neurology, Aarhus University Hospital, invited to participate via e-mail. The headache diagnoses included chronic daily tension-type headaches, migraine, and cluster headaches. All participants gave informed consent prior to their inclusion in the study.

The procedure followed was in accordance with the local ethics standard (The central Denmark Region Committee on Research Ethics) and with the Helsinki Declaration of 1975, as revised in 2000.

### Pain measures and psychological variables

#### Pain measures

Pain intensity and pain unpleasantness were measured with two separate numerical rating scales (NRS) ranging from 0 (“no pain sensation”/“not at all unpleasant”) to 10 (“the most intense pain sensation imaginable”/“the most unpleasant imaginable”) (Price et al., 1994). Pain frequency was assessed with three questions adapted from the Diagnostic Criteria for Temporomandibular Disorders (DC-TMD) (Schiffman et al., 2014). The DC-TMD was chosen as it has previously been used in chronic pain populations and as the validity and reproducibility of the scale is known (Ohrbach et al., 2010; Schiffman et al., 2014). The questions from the DC-TMD were used as a template and we adapted and used the following questions from the DC-TMD:

1. “How often do you experience pain?” Scores ranging from 0 to 4 ([never], [1–6 times a year], [every month], [every week], [every day]).
2. “How long does an average pain episode last?” Scores ranging from 0 to 3 ([less than 30 min per episode], [between 30 min and 2 h per episode], [between 2 h and 7 days per episode], [between 7 days and continuous]).
3. “Over the past 30 days, how many days have you, on average, been in pain?” Scores ranging from 0 to 3 ([less than 1 day], [1 day or more but less than 15 days], [15 days or more but not continuous], [continuous]).

The total pain frequency score was calculated by summing the scores from all three questions, yielding a maximum score of 10.

#### Pain catastrophizing

Pain catastrophizing was assessed using a Danish adaptation of the PCS (Table 1) (Sullivan et al., 1995), which instructs participants to rate a number of specific thoughts and feelings when they experience pain on a 5-point Likert rating scale. The standard instruction is: “In this questionnaire we are interested in the thoughts and feelings you have when you are in pain.” The PCS consists of 13 items with three subscales: rumination (items 8–11), magnification (items 6, 7, 13) and helplessness (items 1–5, 12). The maximum score is 52, with a higher score indicating a higher level of pain catastrophizing. Permission to translate the PCS was obtained from the authors of the original version (Sullivan et al., 1995) (Copy of the English original is available as Supplementary Material). Two independent translations were compared, and inconsistencies were solved by negotiation. The agreed-upon preliminary version was back-translated into English by a person whose mother tongue was English. Inconsistencies were solved by negotiation and final adjustments were made (Behling and Law, 2000).

**Table 1:** **The information given to the participants, including how to rate the statements**.

Pain Catastrophizing Scale for Danish non-clinical and clinical populations.		
Alle oplever smerte på et eller andet tidspunkt i livet. Det kan f.eks. være hovedpine, tandpine og smerter i led og muskler. Vi bliver også udsat for situationer, som kan fremkalde smerter, f.eks. sygdom, skader, tandbehandlinger og operationer. I dette spørgeskema er vi interesseret i tanker og følelser, du har, når du oplever smerter. Nedenfor er der 13 forskellige sætninger, som beskriver forskellige tanker og følelser, som kan være forbundet med smerte. Angiv i hvilken grad du har disse tanker og følelser, når du oplever smerte, ved at skrive det tal, der bedst passer til din oplevelse ud for hver sætning.		
0 = slet ikke, 1 = i ringe grad, 2 = i nogen grad, 3 = i høj grad, 4 = i meget høj grad		
**Nr.**	**Erklæring**	**Score**
1.	Det bekymrer mig hele tiden, om smerterne vil forsvinde.	
2.	Jeg føler, at jeg ikke kan mere.	
3.	Det er frygtelig, og jeg tænker, at det aldrig bliver bedre.	
4.	Det er forfærdeligt, og jeg føler mig overvældet af smerterne.	
5.	Jeg føler, at jeg ikke kan holde det ud længere.	
6.	Jeg bliver bange for at smerterne vil blive værre.	
7.	Jeg tænker hele tiden på andre smertefulde oplevelser.	
8.	Jeg ønsker desperat, at smerten vil forsvinde.	
9.	Jeg kan ikke lade være med at tænke på mine smerter.	
10.	Jeg bliver ved med at tænke på, hvor ondt det gør.	
11.	Jeg bliver ved med at tænke på, hvor meget jeg ønsker, at smerten skal holde op.	
12.	Der er intet jeg kan gøre for at mindske intensiteten af mine smerter.	
13.	Jeg tænker på om der kunne ske noget alvorligt.	

#### Depression

Depressive symptoms were assessed with the Danish version of the Beck Depression Inventory—Second Edition (BDI-II) (Beck et al., 1996). The BDI is a self-report measure that assesses affective/cognitive and somatic aspects of depression over the last 2 weeks. The standard instruction is: “Please choose the statement that best describes how you have felt in the past 2 weeks, including today.” The BDI consists of 21 items, each with four possible responses (0–3) indicating the severity of the symptom. The maximum score is 63, with a higher score indicating a higher level of depressive symptoms. The validity and reliability of the scale has previously been confirmed (Zachariae et al., 2001; Christensen et al., 2009).

#### Anxiety

Anxiety symptoms were measured with the Danish version of the Spielberger State Trait Anxiety Inventory (STAI-Y1, state version) (Spielberger et al., 1983). The STAI-Y1 assesses present cognitive, emotional and behavioral aspects of anxiety. The standard instruction is: “Read every statement and choose the statement that best describes how you feel right now.” The STAI-Y1 consists of 20 items with four possible responses (1–4) to each question. The maximum score is 80, with a higher score indicating a higher level of anxiety. The scale has previously shown acceptable internal consistency in a Danish population (Cronbach's alpha = 0.803) (Vase et al., 2011).

### Statistics

The analysis of the contribution of pain catastrophizing to pain intensity and unpleasantness was conducted in four steps. In the first step, the bivariate associations between pain catastrophizing and pain outcomes (intensity and unpleasantness) were analyzed with unadjusted linear regression analyses together with the contributions of the possible moderator (pain frequency) and covariates (gender, age, anxiety and depressive symptoms). In the second step, the independent contribution of pain catastrophizing to pain outcomes was analyzed with multivariate linear regressions adjusting for the moderator and possible covariates. To avoid overfitting, non-significant covariates identified in the second step were excluded in the third step. To avoid underfitting, the significance level was set at a more liberal10 % level in the first and second steps (Babyak, 2004). In the *third step*, the hypothesis of pain frequency as a moderator of the association between pain catastrophizing and pain outcomes was analyzed with multiple linear regression following previous recommendations (Baron and Kenny, 1986; Aiken and West, 1991). Moderation was considered confirmed if the *interaction term* between the independent variable (PCS) and the moderator (pain frequency) contributed independently over and beyond the contribution of the independent variable and the moderator, while adjusting for covariates that were statistically significantly associated with the pain outcomes of the second step. Adding the interaction term to the equation may introduce considerable multicollinearity, which may lead to problems estimating the regression coefficients. This problem is reduced when the continuous predictors are centered, i.e., variables with a mean of “0” are computed by subtracting the mean of the variable from each value (Neter et al., 1989). All continuous predictors were therefore centered prior to conducting the interaction analysis. The PROCESS macro (www.afhayes.com) was used in the analysis. In the *fourth step*, the interactions for each sample and each pain outcome were probed *post hoc* by examining the simple slopes of the independent variable at specific values of the moderator. The specific values chosen were 1 SD above (high) and 1 SD below (low) the mean. In this analysis, the covariates were omitted to provide a simple visual presentation of the role of the moderator. Statistically significant slopes indicate that pain catastrophizing is significantly associated with the pain outcome at the specific level of the moderator. A significant interaction at step 3 indicates that the slopes are significantly different at different levels of the moderator, when taking the covariates into consideration. The Johnson–Neyman technique was used to identify the level at which the moderator became statistically significant.

Missing items were rare in the dataset (<2%) and appeared to be randomly distributed across cases and were excluded in a pairwise fashion (Little and Rubin, 1987). The statistical analyses were conducted with SPSS, version 19.

For all analyses, *p* < 0.05 was considered significant, with the exception of the first and second steps of the multivariate linear regression analyses where a less conservative *p*-level of 0.10 was considered significant in order to avoid underfitting.

### Assessing the internal consistency and factor structure of the PCS

When assessing internal consistency, an **α** of ≥0.8 for the total PCS and **α**'s of 0.6–0.7 for the subscales were considered acceptable for indicating internal consistency due to the limited number of items on the subscales (Coolican, 1996). Confirmatory factor analysis (CFA) was used to investigate if the previously established factorial structure (Sullivan et al., 1995) would apply to different populations, e.g., Danish non-clinical and clinical pain populations. Results from the maximum likelihood factor dimension reduction, oblimin rotation and a fixed number of factors were used. The fixed number of factors was based on a one-factor structure with all items loading on one common factor, a two-factor structure suggested by Osman et al. (1997), and the original three-factor structure suggested by Sullivan et al. (1995) and further confirmed by other studies of the PCS (Miró et al., 2008; Tremblay et al., 2008; Yap et al., 2008). Several models of CFA were tested to compare models and several fit indices were used to assess and compare model fits, as this strategy overcomes the limitations of each index (Bollen and Long, 1993; Jaccard and Wan, 1996; Marsh et al., 1996). The indices used were: χ^2^ = chi-square test of model fit, the Comparative Fit Index (CFI), the Root Mean Square Error of Approximation (RMSEA) and the Standardized Root Mean Square Residual (SRMR). A statistically significant χ^2^ indicates that a significant proportion of variance within the data is unexplained by the model. CFI values of 0.90 or greater indicate adequate fit. RMSEA values up to 0.08 represent reasonable errors of approximation in the population. SRMR values less than 0.08 indicate a good fit. CFA was tested using Mplus® Version 5 (Munthén and Munthén, 2007).

## Results

### Participants

The total number and gender distribution of the participants are shown in Table 2. Four students were excluded because of incomplete data. The mean age of the non-clinical sample [*M* = 22 (*SD* = 6.2)] was comparable with the mean age of the original sample used to derive the original scale [*M* = 20.1 (*SD* = 5.1)] (Sullivan et al., 1995). In the non-clinical sample, 39% of participants reported that they were experiencing pain at the present time, but their pain intensity scores were low [*M* = 0.80 (*SD* = 1.28)]. Some of the students may have experienced spontaneous pain at the time of assessment but were not excluded from analysis, as they were all confirmed as non-clinical participants. The clinical sample consisted of more than twice as many women as men, and the patients were significantly older (*p* < 0.000) [*M* = 45.9 (*SD* = 15.2)] than the students. Therefore, the subsequent analyses were adjusted for age and gender.

**Table 2:** **Descriptives**.

**Sample**	**Gender**	**Non-clinical**				**Clinical**			
		***N***	**Mean**	***SD***	**Std. Error Mean**	***N***	**Mean**	***SD***	**Std. Error Mean**
Age	Men	118	22.40	7.21	0.66	57	48.79	15.07	2.00
	Women	129	21.71	5.15	0.45	161	44.81	15.17	1.20
Pain Intensity	Men	116	0.94	1.37	0.13	58	3.67	2.65	0.35
	Women	125	0.67	1.18	0.11	160	3.36	2.76	0.22
Pain Unpleasantness	Men	116	1.06	1.48	0.14	58	3.21	2.61	0.34
	Women	125	0.66	1.26	0.11	160	3.20	2.82	0.22
PCS total	Men	118	10.31	6.69	0.62	60	16.90	10.35	1.34
	Women	129	12.26	8.70	0.77	163	22.46	12.02	0.94
BDI total	Men	118	6.14	5.54	0.51	58	12.02	10.71	1.41
	Women	128	5.37	6.37	0.56	160	13.01	9.72	0.77
STAI (State)	Men	118	30.86	7.34	0.68	60	41.85	11.01	1.42
	Women	128	30.62	7.51	0.66	163	41.93	11.42	0.89
Pain frequency	Men	27	3.11	1.85	0.36	58	7.31	2.53	0.33
	Women	124	3.47	1.69	0.15	160	7.07	2.26	0.18

### Pain intensity

#### Non-clinical sample

**Figure 1:**
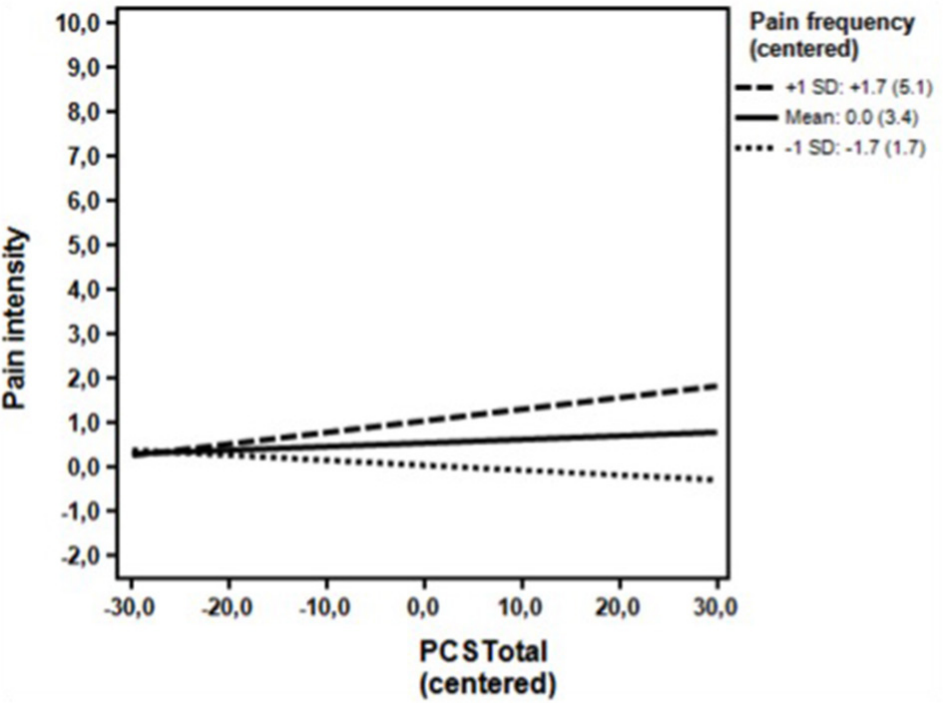
**Non-clinical sample**. Associations between the DV (Pain intensity) and the IV (PCS total - centered) for high (+1 *SD*) levels, moderate level (mean) and low levels (−1 *SD*) of the moderator (Pain frequency - centered) omitting the covariates. The simple regression slope for high (+1 *SD*) was positive and statistically significant (0.03; *p* = 0.02). The simple slopes for moderate levels (mean) (0.01; *p* = 0.468), and low levels (−0.01; *p* = 0.454) were non-significant. *PCS*, Pain Catastrophizing Scale; Numbers in parentheses, Uncentered values of the moderator.

As seen in Table 3, the unadjusted analyses in the first step showed that pain catastrophizing was a statistically significant predictor of pain intensity together with pain frequency and depressive symptoms (BDI) and state anxiety (STAI State anxiety). Neither gender nor age reached statistical significance. In the multivariate analysis at the second analytical step, pain catastrophizing ceased to be a significant predictor when adjusting for the remaining variables. At the third step (Table 3.2), analyzing the possible moderating influence of pain frequency, pain frequency was found to be a significant moderator (*p* = 0.04) of the association of pain catastrophizing with pain intensity, while adjusting for the covariate of the BDI, which was statistically significant (*p* = 0.001) in the second step. The association of pain catastrophizing with pain intensity continued to be statistically non-significant. *Post hoc* probing indicated that higher levels of pain catastrophizing were associated with higher pain intensity however, only at pain frequency value of 6 or higher, corresponding to 17 (6.9%) of the participants (Figure 1).

**Table 3:** **Results of linear regression analyses with pain *intensity* as the dependent variable, pain catastrophizing as the independent variable, and pain frequency as the moderator in a non-clinical sample of 247 healthy volunteers and a clinical sample of 223 patients with chronic headache**.

**DV: Pain intensity**	**Non-clinical sample**				**Clinical sample**			
**3.1.**	**3.1.1. Unadjusted**		**3.1.2. Adjusted**		**3.1.1. Unadjusted**		**3.1.2. Adjusted**	
	**Beta**	***P***	**Beta**	***p***	**Beta**	***p***	**Beta**	***P***
PCS total	0.31	**0.001**	0.05	0.493	0.18	**0.008**	0.07	0.331
Pain frequency	0.48	**0.001**	0.36	**0.001**	0.60	**0.001**	0.55	**0.001**
Gender (female)	−0.11	0.104	0.01	0.933	−0.06	0.423	−0.03	0.635
Age	0.10	0.136	−0.00	0.986	0.04	0.568	0.10	*0.087*
BDI total	0.43	**0.001**	0.28	**0.004**	0.34	**0.001**	0.05	0.528
STAI state anx.	0.30	**0.001**	0.04	0.671	0.33	**0.001**	0.09	0.238
	Adjusted *R*^2^ = 0.29				Adjusted *R*^2^ = 0.39			
**3.2.**			**Coeff.**	***p***			**Coeff.**	***P***
	PCS total		−0.03	0.139	PCS total		0.04	**0.011**
	Pain frequency		0.13	0.067	Pain frequency		0.68	**0.001**
	*PCS × Pain freq.*		0.01	**0.040**	*PCS × Pain freq.*		0.01	**0.017**
	BDI total		0.05	**0.001**	Age		0.01	0.160
	Δ*R*^2^ = 0.02; *p* = 0.04		Adjusted *R*^2^ = 0.34		Δ*R*^2^ = 0.02; *p* = 0.017		Adjusted *R*^2^ = 0.38	

*Table 3.1. Uncentered IV's as predictors of pain intensity in (3.1.1.) unadjusted analyses and (3.1.2) fully adjusted multiple regressions. Table 3.2. Multiple regression analyses including the interaction term between the IV (PCS) and the hypothesized moderator (Pain freq.) with all continuous variables in the model centered to reduce multicollinearity, and adjusting for those covariates (age, gender, BDI, and STAI state anxiety) emerging as statistically significant at the 10% level (at p < 0.1) predictors in the multiple linear regression. Boldface: p < 0.05. Italics: p < 0.10. Abbreviations: IV, Independent variable; DV, dependent variable; PCS, Pain Catastrophizing Scale; BDI, Beck Depression Inventory; STAI, State-Trait Anxiety Inventory; ΔR^2^, R^2^ change due to PCS × Pain freq interaction*.

#### Clinical sample

**Figure 2:**
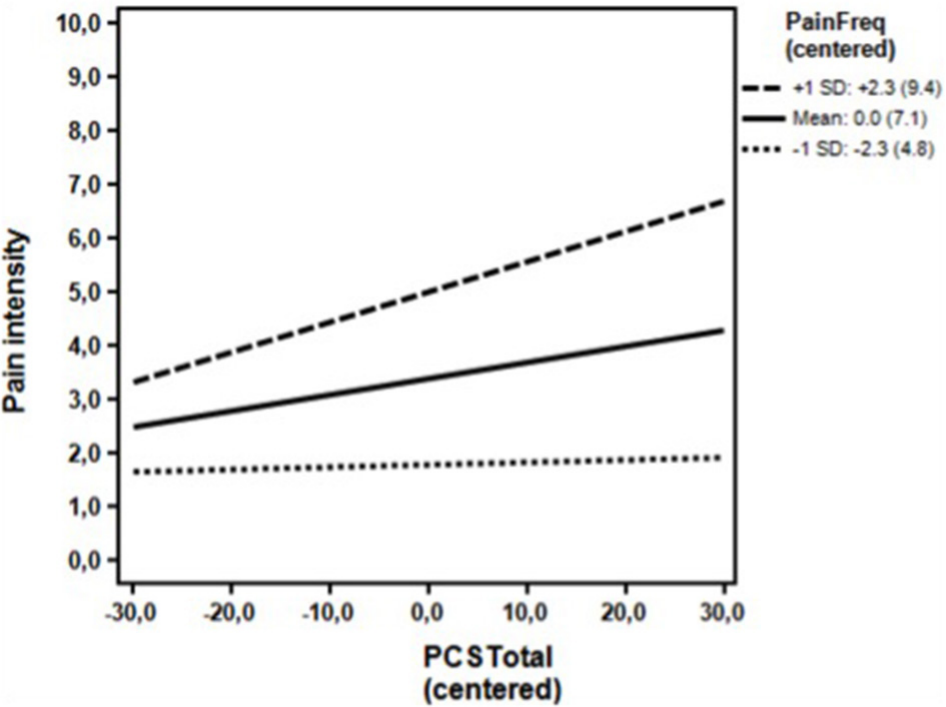
**Clinical sample**. Associations between the DV (Pain intensity) and the IV (PCS total) for high (+1 *SD*) levels, moderate level (mean) and low levels (−1 *SD*) of the moderator (Pain frequency) omitting the covariates. The simple regression slopes for high (0.06; *p* = 0.003) and moderate levels (0.03; *p* = 0.019), were positive and statistically significant. The simple slope for low levels (0.00; *p* = 0.786) was not statistically significant. *PCS*, Pain Catastrophizing Scale; Numbers in parentheses, Un-centered values.

Likewise, as seen in Table 3, the unadjusted analyses in the first step showed that pain catastrophizing was a statistically significant predictor of pain intensity together with pain frequency and depressive symptoms (BDI) and state anxiety (STAI State anxiety). Again, neither gender nor age reached statistical significance. In the multivariate analysis at the second analytical step, only pain frequency (*p* < 0.001) and age (*p* = 0.087) reached statistical significance at the 10% level (see methods section), when adjusting for the remaining variables. At the third step (Table 3.2), analyzing the possible moderating influence of pain frequency, pain frequency was found to be a significant moderator (*p* = 0.017) of the association between pain catastrophizing and pain intensity, while adjusting for age, which reached statistical significance at the 10% level in the second step. The association between pain catastrophizing and pain intensity now reached statistical significance (*p* = 0.011). *Post hoc* probing indicated that higher levels of pain catastrophizing were associated with higher pain intensity at both moderate and high levels of the pain frequency. The value of the pain frequency, at which it reached statistical significance was 6 or higher, corresponding to 160 (73.7%) of the patients. The association was not statistically significant at low levels of the moderator (Figure 2).

### Pain unpleasantness

#### Non-clinical sample

**Figure 3:**
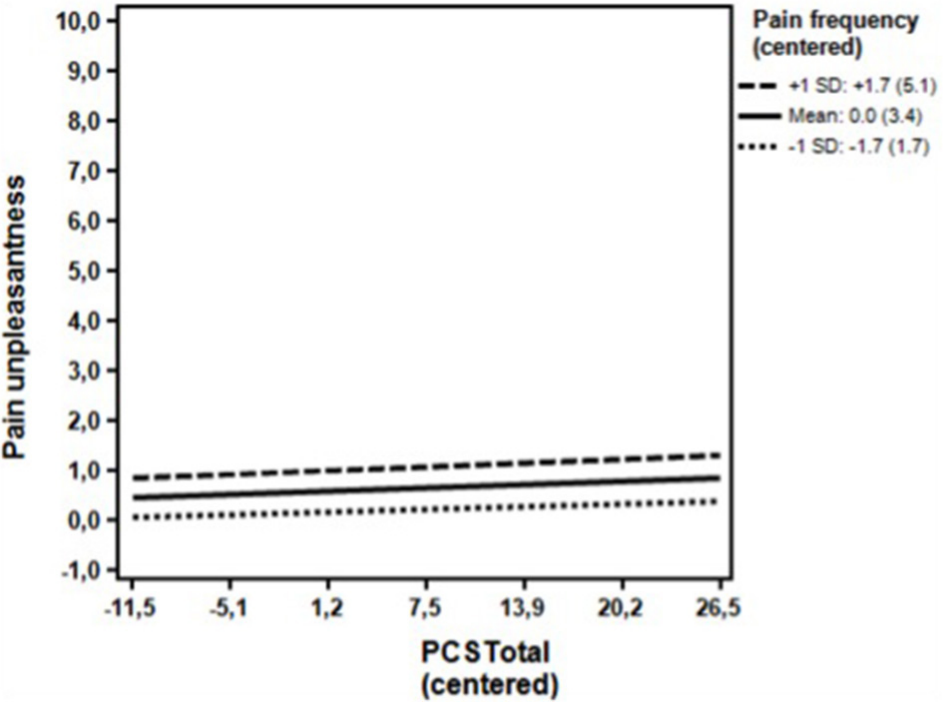
**Non-clinical sample**. Associations between the DV (Pain unpleasantness) and the IV (PCS total) for high (+1 *SD*) levels, moderate level (mean) and low levels (−1 *SD*) of the moderator (Pain frequency) omitting the covariates. The simple regression slopes for high (0.01; *p* = 0.324), moderate levels (0.01; *p* = 0.369), and low levels (0.01; *p* = 0.612) were all statistically non-significant. *PCS*, Pain Catastrophizing Scale; Numbers in parentheses, Un-centered values.

As seen in Table 4, the unadjusted analyses in the first step showed that pain catastrophizing was a statistically significant predictor of pain unpleasantness together with pain frequency, gender, age, depressive symptoms (BDI) and state anxiety (STAI State anxiety). In the multivariate analysis at the second analytical step, pain catastrophizing ceased to be a significant predictor when adjusting for the remaining variables, with only pain frequency and depressive symptoms (BDI) being statistically significantly associated with pain unpleasantness. At the third step (Table 4.2), analysing the possible moderating influence of pain frequency, pain frequency was not found to moderate the association of pain catastrophizing with pain unpleasantness, while adjusting for the covariate of BDI, and the association of pain catastrophizing with pain intensity continued to be statistically non-significant. *Post hoc* probing showed that pain catastrophizing was not associated with pain unpleasantness at all levels of pain frequency (Figure 3)

**Table 4:** **Results of linear regressions with pain *unpleasantness* as the dependent variable, pain catastrophizing as the independent variable, and pain frequency as the moderator in a non-clinical sample of 247 healthy volunteers and a clinical sample of 223 patients with chronic headache**.

**DV: Pain unpleasant.**	**Non-clinical sample**				**Clinical sample**			
**4.1.**	**4.1.1. Unadjusted**		**4.1.2. Adjusted**		**4.1.1.Unadjusted**		**4.1.2. Adjusted**	
	**Beta**	***P***	**Beta**	***p***	**Beta**	***p***	**Beta**	***p***
PCS total	0.28	**0.001**	0.04	0.631	0.25	**0.001**	0.13	*0.067*
Pain frequency	0.38	**0.001**	0.30	**0.001**	0.59	**0.001**	0.53	**0.001**
Gender (female)	−0.14	**0.026**	0.03	0.709	−0.00	0.980	0.01	0.836
Age	0.15	**0.018**	0.02	0.819	0.02	0.811	0.10	*0.090*
BDI total	0.33	**0.001**	0.19	**0.064**	0.36	**0.001**	−0.01	0.873
STAI state anx.	0.20	**0.002**	0.01	0.927	0.39	**0.001**	0.19	**0.018**
	Adjusted *R*^2^ = 0.15				Adjusted *R*^2^ = 0.41			
**4.2.**			**Coeff.**	***p***			**Coeff.**	***p***
	PCS total		0.01	0.802	PCS total		0.04	**0.006**
	Pain frequency		0.20	**0.013**	Pain frequency		0.62	**0.001**
	*PCS × Pain freq.*		0.00	0.987	*PCS × Pain freq.*		0.02	**0.001**
	BDI total		0.04	**0.017**	Age		0.02	0.081
					STAI state anx.		0.04	**0.026**
	Δ*R*^2^ = 0.0, *p* = 0.99		Adjusted *R*^2^ = 0.18		Δ*R*^2^ = 0.04, *p* < 0.001		Adjusted *R*^2^ = 0.44	

*Table 4.1. Uncentered IV's as predictors of pain unpleasantness in (4.1.1) unadjusted analyses and (4.1.2) fully adjusted multiple regressions. Table 4.2. Multiple regression analyses including the interaction term between the IV (PCS) and the hypothesized moderator (Pain freq.) with all continuous variables in the model centered to reduce multicollinearity, and adjusting for those covariates (age, gender, BDI, and STAI state anxiety) emerging as statistically significant at the 10% level (p < 0.10) in the multiple linear regression (2a). Boldface: p < 0.05. Italics: p < 0.10. Abbreviations: IV, Independent variable; DV, dependent variable; PCS, Pain Catastrophizing Scale; BDI, Beck Depression Inventory; STAI, State-Trait Anxiety Inventory; ΔR^2^, R^2^ change due to PCS × Pain freq interaction*.

#### Clinical sample

**Figure 4:**
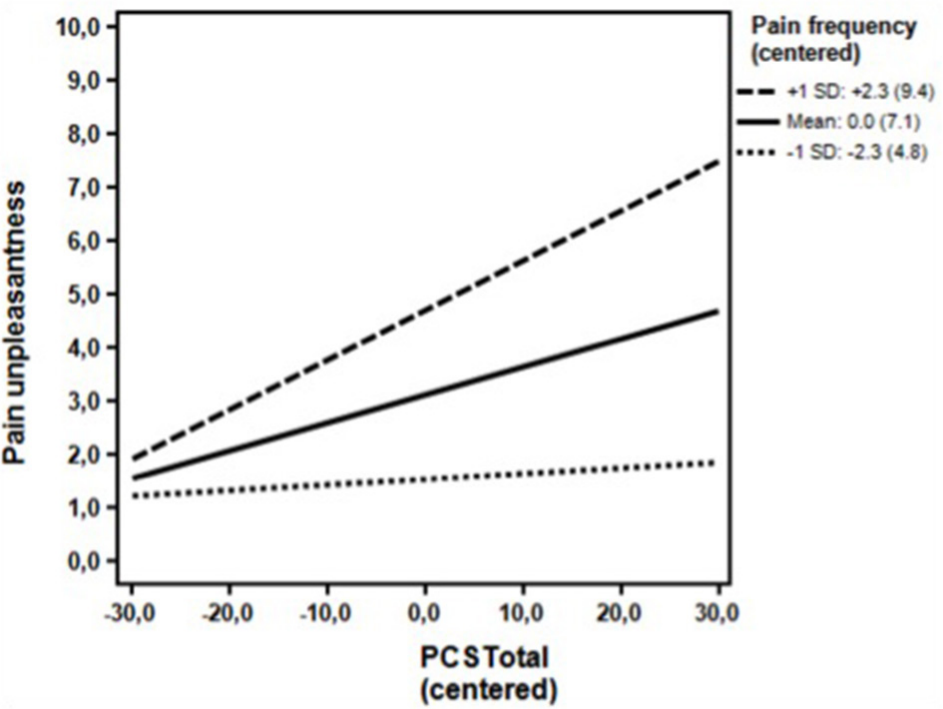
**Clinical sample**. Associations between the DV (Pain unpleasantness) and the IV (PCS total) for high (+1 *SD*) levels, moderate level (mean) and low levels (−1 *SD*) of the moderator (Pain frequency) omitting the covariates. The simple regression slopes for high (0.09; *p* < 0.001) and moderate levels (0.05; *p* < 0.001) were positive and statistically significant. The simple slope for low levels of the moderator (0.01; *p* = 0.486) was statistically non-significant. *PCS*, Pain Catastrophizing Scale; Numbers in parentheses, Un-centered values.

As seen in Table 4, the unadjusted analyses in the first step showed that pain catastrophizing was a statistically significant predictor of pain unpleasantness together with pain frequency and depressive symptoms (BDI) and state anxiety (STAI State anxiety). Neither gender nor age reached statistical significance at the 10% level. In the multivariate analysis at the second analytical step, pain catastrophizing, age, and state anxiety were significant predictors at the 10% level when adjusting for the remaining variables. At the third step (Table 4.2), analyzing the possible moderating influence of pain frequency, pain frequency was found to be a significant (*p* < 0.001) moderator of the association of pain catastrophizing with pain unpleasantness, while adjusting for the covariates of State anxiety and age. The association of pain catastrophizing with pain unpleasantness now emerged as statistically significant (*p* = 0.006). *Post hoc* probing indicated that higher levels of pain catastrophizing were associated with higher pain unpleasantness at both high and moderate levels of the pain frequency. A pain frequency value of 6 or higher indicated the level at which the moderator became statistical significant. A total of 160 (73.7%) had pain frequency scores of 6 or higher. The association was not statistically significant at low levels of the moderator (Figure 4).

### Psychometric properties of the danish adaptation of the PCS

The internal consistencies were acceptable for both samples. In the non-clinical sample, Cronbach's alpha's were 0.91 (total PCS), 0.90 (rumination), 0.71 (magnification), and 0.85 (helplessness). In the clinical sample, Cronbach's alphas were 0.94 (total PCS), 0.91 (rumination), 0.70 (magnification), and 0.91 (helplessness). As seen in Table 5, CFA generally showed the best fit for the three-factor structure. CFI was only acceptable for the three-factor model in the non-clinical sample and for both the two and the three-factor model in the clinical sample. While the RMSEA was only acceptable for the three-factor structure in the clinical sample, the three-factor structure also had an overall better fit than the one- and two-factor models for the non-clinical sample. The SRMR was acceptable for all of the three models tested, but showed a better fit for the three-factor model.

**Table 5:** **CFA for the PCS in non-clinical and clinical samples**.

	**ML χ^2^**	**χ^2^/df**	**SCF**	**CFI**	**RMSEA**	**SRMR**
**NON-CLINICAL SAMPLE**						
Null	1484.541^**^ (78)	19.03				
1 factor (13 items)	332.572^**^ (65)	5.11	1.313	0.810	0.129	0.071
2 factor (6 + 7 items)	305.296^**^ (64)	4.77	1.309	0.828	0.124	0.076
3 factor (3 + 4 + 6 items)	162.148^**^ (62)	**2.62**	**1.299**	**0.929**	**0.081**	**0.054**
**CLINICAL SAMPLE**						
Null	1924.548^**^ (78)	24.67				
1 factor (13 items)	274.272^**^ (65)	4.22	1.201	0.887	0.120	0.052
2 factor (6 + 7 items)	240.717^**^ (64)	3.76	1.192	0.904	0.111	0.053
3 factor (3 + 4 + 6 items)	146.896^**^ (62)	**2.37**	**1.209**	**0.954**	**0.078**	**0.045**

*Bold indicates best fit for the sample. All χ^2^ analyses were statistically significant*.

** *P < 0.0000; ML, Maximum likelihood; χ^2^, chi-square test of model fit; df, degrees of freedom in parentheses, SCF, scale correction factor, CFI, comparative fit index; RMSEA, Root mean square error of approximation; SRMR, standardized root mean square residual; Two-factor model, After Osman et al. (1997) (HELPMAG 1,2,3,4,6,7,13 + RUM 5,8,9,10,11,12)*.

*Three-factor, Sullivan et al. (1995) (HELP 1,2,3,4,5,12 + MAG 6,7,13 + RUM 8,9,10,11)*.

## Discussion

When analyzing the possible moderating role of pain frequency, the association between pain catastrophizing and pain intensity was shown to be moderated by levels of pain frequency in both the clinical and the non-clinical sample. Our results from the unadjusted bivariate analyses revealed that pain catastrophizing was statistically associated with both pain intensity and pain unpleasantness in both samples. When adjusting for depressive symptoms and state anxiety in multivariate analyses, pain catastrophizing ceased to be a statistically significant predictor at the 5% significance level for both pain intensity and unpleasantness in both samples. When analyzing the possible moderating role of pain frequency, the association between pain catastrophizing and pain intensity was moderated by the level of pain frequency in both samples. *Post hoc* probing showed that the moderator reached statistical significance at pain frequency levels of 6 or higher for both samples. In the clinical sample, this cutoff corresponded to 160 (73.7%) of the patients, while in the non-clinical sample; only 17 (6.9%) reported a pain frequency of 6 or higher. For pain unpleasantness, a moderating effect of pain frequency was only found in the clinical sample, again with the moderator reaching statistical significance at pain frequency levels of 6 or higher.

To our knowledge, this is the first study to show that pain frequency moderates the relationship between pain catastrophizing and pain in non-clinical and clinical populations. The potential mechanisms underlying these findings will be discussed. Furthermore, the results of the study supported the psychometric properties of the Danish adaptation of the PCS in both a non-clinical and a clinical sample.

### Pain frequency and potential underlying mechanisms

The finding that high levels of pain frequency moderated the association between pain catastrophizing and pain intensity and unpleasantness, i.e., independently of anxiety and depressive symptoms, in both non-clinical and clinical populations suggests that pain frequency may be central to the understanding of the relationship between pain catastrophizing and pain.

As mentioned in the introduction, previous studies have typically found an association between pain catastrophizing and pain when the relationship was not adjusted for anxiety and depression (Beneciuk et al., 2010; Vranceanu et al., 2010; Lucey et al., 2011). However, once the relationship is adjusted for anxiety and depression, the results are mixed. These findings are similar to the results of the present study showing a significant relationship between pain catastrophizing and pain when the association was not adjusted for anxiety and depression. Importantly, when pain frequency was taken into account, pain catastrophizing remained significantly related to pain independently of anxiety and depression. The finding that high levels of pain frequency moderate the relationship between pain catastrophizing and pain suggests that pain frequency may be pivotal to understanding the apparently mixed results of the relationship between pain catastrophizing and pain when adjusting for overlapping psychological constructs such as anxiety and depression.

This point of view could be seen as strengthened by the differences observed between the clinical and the non-clinical sample. When pain frequency was low, as seen in the non-clinical sample, the strength of the relationship between pain frequency and pain intensity and unpleasantness was also reduced, limiting the moderating effect of pain frequency (Tables 3, 4). Yet, despite the difference in the number of participants who reached a relevant level of pain frequency in the clinical vs. the non-clinical population, it is worth noting that the level of pain frequency at which pain catastrophizing exerted a moderating effect on pain was the same in both groups (6 or higher).

The importance of frequency and the possible underlying mechanisms are also illustrated in longitudinal studies of chronic tension type headache showing that a high frequency of pain leads to increased tenderness and eventually to lowered pain thresholds, thereby supporting the hypothesis that a high frequency of pain leads to sensitization of the nociceptive system (Buchgreitz et al., 2008). Also, cross-sectional population-based studies of headache have shown that the higher the pain frequency, the higher the odds ratio for anxiety, and depression independently of the exact diagnosis of headache (migraine vs. non-migrainous headache) (Zwart et al., 2003). These findings are in agreement with studies indicating that supraspinal sensitivity and attention to and vigilance toward pain may contribute to the activation of pain networks (Bendtsen et al., 1996; Cathcart et al., 2010; Bezov et al., 2011).

Taken together, these findings suggest that a high frequency of pain is associated with increased sensitization, which has been shown to activate a number of supraspinal structures like thalamus, somatosensory cortices and the anterior cingulate cortex (Staud et al., 2007; Cathcart et al., 2010; Bezov et al., 2011). This activation may in itself lead to supraspinal sensitization with increased attention to pain, increased hypervigilance, and emotional distress and hence increased levels of pain catastrophizing (Cathcart et al., 2010; Bezov et al., 2011; Vase et al., 2011). However, pain catastrophizing has also in itself been related to increased activity in areas such as the anterior cingulate cortex and the insula and decreased activity in prefrontal areas, thereby suggesting that pain catastrophizing may be a form of cognitive-emotional sensitization that facilitates nociceptive processing and/or inhibits top-down modulation of pain (Gracely et al., 2004; Seminowicz and Davis, 2007; Cathcart et al., 2010; Vase et al., 2012). Based on cross-sectional studies like the present, it is not possible to deduce whether a high frequency of pain is implicated in pain causing pain catastrophizing or vice versa, but once these processes are activated, they are likely to reinforce each other. Such reinforcement could involve the anterior cingulate cortex as it has been shown to be involved in the integration of physical, emotional, and cognitive aspects of pain and distress (Staud et al., 2007). This hypothesis needs to be tested further in experimental studies that manipulate pain frequency and measure the psycho-neurophysiological underpinnings of pain catastrophizing. Nevertheless, the present study suggests that it is important to incorporate measures of pain frequency in future studies as it may be central to the understanding of the relationship between the tendency to catastrophize and the experience of pain.

### Validation of the danish version of the pain catastrophizing scale

An acceptable internal consistency of the PCS was found for the total scores and subscales for both the non-clinical and the clinical sample. The magnification scale had lower internal consistency than the other subscales. Studies following the work by Sullivan have shown that a low internal consistency for the magnification subscale may be explained by the low number of items (Sullivan et al., 1995; Osman et al., 2000; Miró et al., 2008).

CFA showed acceptable fit in the data for both samples, which is in accordance with previous findings and; overall, the three-factor structure was the best fit for both samples as demonstrated in previous studies (Van Damme et al., 2002; Crombez et al., 2003; Miró et al., 2008; Tremblay et al., 2008). The Danish version of the PCS is therefore considered valid for use in both non-clinical and clinical populations.

## Conclusions and implications

The present study shows that pain frequency may be central to the unique contribution of pain catastrophizing, independently of anxiety and depressive symptoms to pain in non-clinical and clinical pain populations. In future studies it may therefore be helpful to consider pain frequency when investigating the relationship between pain catastrophizing and pain.

## Limitations

Two different clinical pain samples were included in the study. One of the aims of the present study was to validate the PCS in a Danish population. In order to ensure a sufficiently large clinical population we included both rheumatoid arthritis and headache patients, which furthermore allowed us to explore the properties of the PCS across different clinical populations. While this could potentially introduce further variation, the pilot testing indicated that the two clinical groups had similar levels of pain intensity and unpleasantness, and it was therefore decided to combine the two groups in the present study. Furthermore, the final results revealed no significant difference between headache and arthritis patients in pain intensity (*p* = 0.109) or pain unpleasantness (*p* = 0.092), suggesting that the two clinical groups were comparable with respects to the variables investigated in the present study.

The influence of pain catastrophizing was adjusted for depression using the BDI. It is important to be aware that the BDI has items that may capture somatic content, which could give reason to a misleading impression of an affective disturbance in clinical vs. non-clinical populations. However, when we directly tested the differences in the BDI scores in the clinical vs. non-clinical populations with and without these items, no significant differences for the two populations were found. Thus, we do not believe that this influences the overall findings of the study.

It cannot be precluded that the relationship between pain catastrophizing and pain could be influenced by additional psychological factors such as anger or fear. However, so far there has been precedence for controlling the relationship between pain catastrophizing and pain for anxiety and depression (Vase et al., 2011; Khan et al., 2012; Wade et al., 2012) and therefore this approach was chosen.

At present there is no stand-alone measure for pain frequency and therefore we chose to measure pain frequency using items from the Research Diagnostic Criteria for Temporomandibular Disorder as this offered a clinically relevant and well-validated measure of frequency. It is, however, important to be aware that the questionnaire also includes questions that relate to pain duration, and it does not standardize the outcome scores as seen in more recent questionnaires investigating frequency-duration-severity as a composite measure (Salamon et al., 2014). Still, our study is, to the best of our knowledge, the first to explore the role of pain frequency in the association between pain catastrophizing and pain using a clinically relevant measure of pain frequency and to show that this parameter is important in understanding the relationship between pain catastrophizing.

## Author contributions

**Table:** 

**Author**	**Conception**	**Design**	**Data acquisition**	**Analysis and interpretation of data**	**Revision of intellectual content**	**Final approval of version to be published**
Heidi Kjøgx	X	X	X	X	X	X
Robert Zachariae	X	X		X	X	X
Mogens Pfeiffer-Jensen			X	X	X	X
Helge Kasch			X	X	X	X
Peter Svensson	X	X		X	X	X
Troels S. Jensen	X	X		X	X	X
Lene Vase	X	X		X	X	X

### Conflict of interest statement

The authors declare that the research was conducted in the absence of any commercial or financial relationships that could be construed as a potential conflict of interest.
