# Supplementary material for: Pain frequency moderates the relationship between pain catastrophizing and pain
Source: Front Psychol. 2014 Dec 19;5:1421. doi: 10.3389/fpsyg.2014.01421 (PMC4297917; doi:10.3389/fpsyg.2014.01421)
Supplement: Supplementary file 1 [file DataSheet1.PDF]

Ψ

Copyright © 1995
Michael JL Sullivan

PCS-EN

Client No.: __________ Age: _____ Sex: M(__) F(__) Date: _______________

Everyone experiences painful situations at some point in their lives. Such experiences may include headaches, tooth pain, joint or muscle pain. People are often exposed to situations that may cause pain such as illness, injury, dental procedures or surgery.

We are interested in the types of thoughts and feelings that you have when you are in pain. Listed below are thirteen statements describing different thoughts and feelings that may be associated with pain. Using the following scale, please indicate the degree to which you have these thoughts and feelings when you are experiencing pain.

0 – not at all **1** – to a slight degree **2** – to a moderate degree **3** – to a great degree **4** – all the time

When I'm in pain …

1 ☐ I worry all the time about whether the pain will end.

2 ☐ I feel I can't go on.

3 ☐ It's terrible and I think it's never going to get any better.

4 ☐ It's awful and I feel that it overwhelms me.

5 ☐ I feel I can't stand it anymore.

6 ☐ I become afraid that the pain will get worse.

7 ☐ I keep thinking of other painful events.

8 ☐ I anxiously want the pain to go away.

9 ☐ I can't seem to keep it out of my mind.

10 ☐ I keep thinking about how much it hurts.

11 ☐ I keep thinking about how badly I want the pain to stop.

12 ☐ There's nothing I can do to reduce the intensity of the pain.

13 ☐ I wonder whether something serious may happen.

…Total
